# An ancestral variant causing type I xanthinuria in Turkmen and Arab families is predicted to prevail in the Afro‐Asian stone‐forming belt

**DOI:** 10.1002/jmd2.12077

**Published:** 2019-12-05

**Authors:** Hava Peretz, Michael Korostishevsky, David M. Steinberg, Mustafa Kabha, Sali Usher, Irit Krause, Hannah Shalev, Daniel Landau, David Levartovsky

**Affiliations:** ^1^ Clinical Biochemistry Laboratory Sourasky Medical Center Tel Aviv Israel; ^2^ Human Molecular Genetics and Biochemistry, Sackler School of Medicine Tel Aviv University Tel Aviv Israel; ^3^ Department of Anatomy and Anthropology, Sackler School of Medicine Tel Aviv University Tel Aviv Israel; ^4^ Department of Statistics and Operations Research Tel Aviv University Tel Aviv Israel; ^5^ Department of History, Philosophy and Judaic Studies The Open University of Israel Ranana Israel; ^6^ Department of Pediatrics C, Schneider Children's Medical Center, Petach Tikva, Sackler School of Medicine Tel Aviv University Tel Aviv Israel; ^7^ Department of Pediatrics, Soroka Medical Center Ben Gurion University of the Negev Beer Sheva Israel; ^8^ Department of Pediatrics B, Schneider Children's Medical Center, Sackler School of Medicine Tel Aviv University Tel Aviv Israel; ^9^ Department of Rheumatology, Tel Aviv Sourasky Medical Center Sackler School of Medicine, Tel Aviv University Tel Aviv Israel

**Keywords:** Afro‐Asian stone‐forming belt, ancestral mutation, Arabs, genealogical history, time to most recent common ancestor, Turkmens, xanthinuria, *XDH* gene

## Abstract

Classical xanthinuria is a rare autosomal recessive metabolic disorder characterized by lack of xanthine dehydrogenase activity that often manifests as xanthine urolithiasis and risk of drug toxicity. Variants in the *XDH* or *HMCS* gene underlie classical xanthinuria type I and type II, respectively. Here we present two Israeli Arab families affected by type I xanthinuria in whom a c.2164A>T (Lys722Ter) variant in the *XDH* gene, previously reported in a Turkish family of Turkmen origin, was identified. Analysis of polymorphic markers surrounding the variant site revealed common haplotypes spanning 0.6 Mbp shared by all three, and 1.7 Mbp shared by two of the studied families. By applying Bayesian methods to a simple model of crossover events through generations in the chromosomes carrying the variant, the most recent common ancestor of these families was found to be 179 (95% credible limit 70) generations old. The estimated antiquity of the variant, the historical genealogy of the affected families and the history and present day dispersion of their people strongly suggest prevalence of this variant in the Afro‐Asian stone‐forming belt. As far as we are aware, this is a first report of an ancient variant causing xanthinuria with potential wide geographical dispersion.

**SYNOPSIS**

This work presents evidence for an ancient founder variant causing type 1 classical xanthinuria in apparently unrelated Turkmen and Arab families; it raises the awareness to its potential presence in regions of the Afro‐Asian stone‐forming belt and may help identification of new cases and prevention of significant adverse clinical and pharmacological implications.

## 1 INTRODUCTION

Classical xanthinuria is a rare autosomal recessive metabolic disorder due to impaired xanthine dehydrogenase (XDH) activity resulting in lack of uric acid (UA) formation and accumulation of its precursors xanthine and hypoxanthine. Type I xanthinuria (MIM 278300) is caused by variants in the *XDH* gene^1^ and results in isolated XDH deficiency. In type II xanthinuria (MIM 603592) variants in the human molybdenum cofactor sulfurase gene (*HMCS*) cause combined XDH and aldehyde oxidase (AO) deficiency.^2^ The clinical manifestations of the two types of xanthinuria are indistinguishable, the most prominent being formation of xanthine calculi in the urinary tract in about 40% of the affected individuals.^3^ Both XDH and AO are drug‐metabolizing enzymes^4^ and their absence may lead to drug induced toxicity. Interestingly, more than 2/3 of the xanthinuria cases have been reported from Mediterranean and Middle Eastern countries,^3^ a region that belongs to the Afro‐Asian stone‐forming belt.^5^ In Israel we encountered five Jewish and eight Arab families affected by classical xanthinuria (Peretz et al., unpublished data).^6^, ^7^ Among them, in two apparently unrelated Arab families we identified a c.2164A>T (Lys722Ter) variant in the *XDH* gene that was previously reported in a Turkish family.^8^ The aim of the present work was to find out whether this variant might be an ancient variant that is widely dispersed in Afro‐Asian regions prone to urolithiasis.

## 2 METHODS

### 2.1 Patients and control subjects

**Figure 1:**
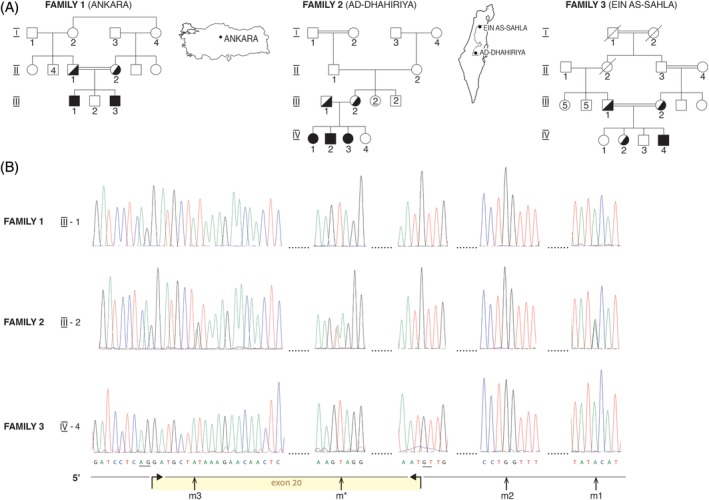
Geographic origin, pedigrees (A) and sequence chromatograms showing the c.2164A>T variant and three associated SNPs in the *XDH* gene in members of the affected families. A, In families 1 and 3 the current residence is shown, whereas in family 2, the mother's native origin is indicated. The geographic maps were adapted from https://commons.wikimedia.org/w/index.php?curid=6064736. Conventional genetic symbols are used to depict family pedigrees. B, Below the chromatograms the horizontal arrows demarcate the 5′ (c.2101) and 3′ (c.2197) ends of exon 20 of the *XDH* gene. The acceptor AG and donor GT intronic splice sites are underlined. The vertical arrows point to the mutation and SNPs sites. Note that the patients in families 1 and 3 are homozygous TT for the c.2164A>T variant and homozygous AA, GG and AA for the m3 (c.2107A/G), m2 (c.2197+42G/C) and m1 (c.2197+68G/A) polymorphisms, whereas the carrier mother in family 2 is heterozygous AT for the variant and heterozygous AG, homozygous GG and heterozygous AG, for the respective polymorphisms

Three families with six affected individuals were studied (pedigrees depicted in Figure 1A). Eleven DNA samples of members of these families and 28 control DNA samples from four apparently unrelated Israeli Arab families were analyzed.

### 2.2 DNA analyses

DNA was manually extracted from peripheral blood samples.^6^ The c.2164A>T variant (**m**) and three single nucleotide polymorphisms (SNPs) designated m1, m2 and m3 were analyzed by PCR amplification followed by Sanger sequencing (Hylabs, Rechovot, Israel) (Table S1, Figure 1B). Microsatellite polymorphic markers designated M1‐M10, within and in the vicinity of the *XDH* gene were analyzed by PCR (Table S1) and validated by Sanger sequencing. For fragment length evaluation PCR was performed using 5′‐6FAM labeled forward primers followed by separation of PCR products on a 3130xl Genetic Analyzer (Applied Biosystems) according to manufacturer's instructions (Figure S1A). The apparent allele sizes slightly differed from the actual number of bp obtained by Sanger sequencing yet the observed alleles were consistent with Mendelian segregation in the studied families (Figure S1B) and thus haplotypes associated with variant and control chromosomes were derived accordingly.

### 2.3 Estimation of the time to the most recent common ancestor

For estimation of the time to the most recent common ancestor (MRCA) Bayesian methods were applied to a simple model of crossover events through generations in the chromosomes carrying the inherited variant. The model relates the length *U* (in Morgans) of the intact interval (on one side of the variant site) to the number *T* of generations between the common ancestor and the recently selected carriers (detailed description of the method is presented in Supporting Information).

### 2.4 Genealogical history of the affected families

The method of “networking” between families was employed^9^ using several sources of information. On the study of Arab families extensive literature consisting of files containing thousands of family trees, divided into “Qays” and “Yaman” or “the northern tribes” and “the southern tribes” and other subdivisions was consulted.^10^ Information on the migration routes of the families and tribes throughout the entire Arab region and marriage ties with other families as well as how new branches were formed with new names was extracted from records of the Muslim Shari'a court and the Ottoman land registry authorities in Jenin and Nablus. Documents belonging to the families, as well as memoirs and biographies of prominent members of these families and studies on various social aspects served as additional source for studying the families' “networking.”^11^, ^12^, ^13^

## 3 RESULTS

### 3.1 Demographic, clinical, and molecular findings

Six affected individuals were identified in three families.

Family 1, residing in Ankara, Turkey is of Turkmen origin and was previously described in detail.^8^ Two symptomatic brothers of consanguineous parents (Figure 1A) presented with renal calculi and were homozygous for the c.2164A>T (Lys722Ter) variant in the *XDH* gene.

Family 2 is part of an extended Bedouin‐Arab pedigree (not shown) residing in the Negev in Southern Israel. Three affected children were identified in this family. The mother was born in ad‐Dhahiriya south‐west to Hebron in the West Bank of the Jordan River (Figure 1A). The index case (IV‐1), a young girl, was followed for a neurogenic bladder and was asymptomatically found to have undetectable levels of UA in serum and urine. Consequently, two older asymptomatic siblings were also found to have zero levels of serum and urinary UA. Renal ultrasound did not reveal any stones in the three patients. An additional sibling had normal levels of UA. The mother is carrier of the c.2164A>T (Lys722Ter) variant (Figure 1B), the father is carrier of another variant in the *XDH* gene (Peretz et al., unpublished data) and the affected children are compound heterozygotes (Figure S1B).

Family 3 is an Arab family living in the 'Ayn al‐Sahala village, located in the Wadi 'ara area in Northern Israel. The parents of the affected child are cousins (Figure 1A). He first presented at the age of 5.5 years with abdominal pain and ultrasound scanning of the abdomen revealed a small sized kidney without hydronephrosis. At the age of 7 years the boy had an episode of urinary retention and an obstructing stone was identified in the urethral meatus. The stone was extracted. Four years later a dimercaptosuccinic acid (DMSA) scan showed a hypoplastic left kidney and a compensatory hypertrophy of the right kidney. Since the left kidney contributed only 7% to the total kidney function and because of the past history of urolithiasis, left nephrectomy was performed. Classical xanthinuria was suspected based on undetectable UA levels in blood and 0.2 mg/dl UA in urine. No additional episodes of urolithiasis were reported. Recent levels of serum creatinine (10/2017) were within normal limits (0.8 mg/dl), microalbuminuria (90 μg/mg creatinine, normal <30) was present. The affected child is homozygous for the c.2164A>T (Lys722Ter) variant in the *XDH* gene whereas the parents and one of the unaffected siblings are carriers (Figure 1).

### 3.2 Identification of progenitor haplotypes

**Figure 2:**
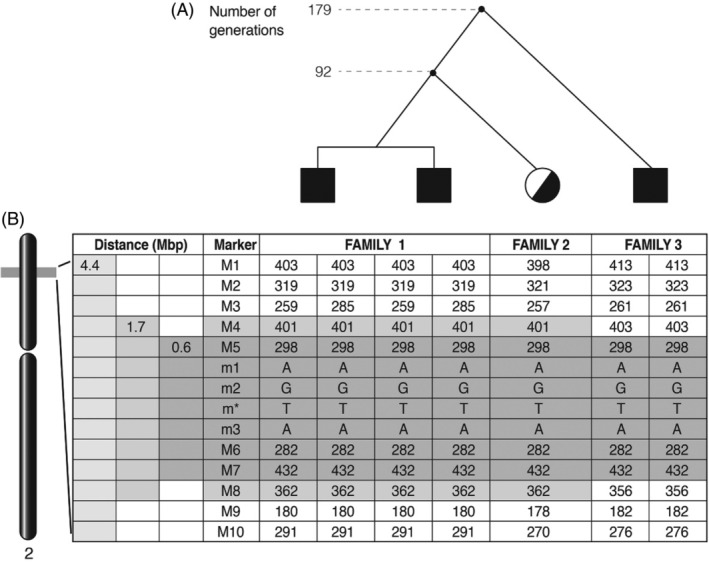
Proposed genealogical tree and estimated times to MRCAs (A) based on shared haplotypes linked to the *XDH* gene c.2164A>T variant (B) in families 1, 2, and 3. A, The proposed genealogical tree with two knots linking the three families and the estimated number of generations to the MRCAs. B, The variant (m*) and the haplotypes of 13 polymorphic markers surrounding the mutation, encompassing a distance of 4.4 Mbp on the short arm of chromosome 2 (2p22‐23) in members of the three affected families is shown. Putative progenitor haplotypes defined by the conserved alleles at distances of 1.7 and 0.6 Mbp, respectively, are highlighted. Note that both affected children in family 1 are heterozygous for marker M3, yet homozygous for the more distant markers M2 and M1 suggesting a de novo variant in marker M3 in a former generation rather than a recombination event

Segregation analysis of 13 polymorphic markers in the affected families revealed conserved alleles of six markers linked to the c.2164A>T variant that construct a putative progenitor haplotype spanning 0.6 Mbp. An extended shared haplotype of eight markers spanning 1.7 Mbp was identified in families 1 and 2 (Figure 2B). The distribution and frequencies of the polymorphic alleles in control chromosomes is shown in Table S2. In 28 independent control chromosomes 27 different haplotypes were discerned, none of which were the putative progenitor haplotype (not shown).

### 3.3 Time to the MRCA

The intervals in Morgan units between the variant and the polymorphic markers used in the computation of the MRCAs are given in Table S3. Based on this data, the estimated distance between all three families is 179 generations (95% credible limit 70 generations); and the estimated distance between families 1 and 2 is 92 generations (95% credible limit 37 generations). The credible limits are quite robust with respect to the upper limit of the prior distribution.

### 3.4 Genealogic history of the affected families

Family 1 is of Turkmen origin and belongs to the Kaya (Qayi) tribe. According to the family tree (Figure 1A) the grandmother sisters are the most probable source of the variant in this family and we assume that they also originate from the Qayi tribe or from a historically closely related tribe. The Qayi tribe belongs to the “Ughuz” coalition that affiliates itself with the Seljuks, the origin of the Ottoman dynasty. Branches of this coalition settled in Palestine (particularly in the western margins of the Jezreel Valley, the region of Karkur‐Caesarea and in the Hebron/Al Khalil region) starting from the 12th century under the Ayyubid (UNESCO document^14^), Mameluke and Ottoman rules. Some of their members occupied senior military and administrative positions and became well known, such as the al‐Askari, al‐Jundi, and Churbaji families. The large majority underwent a process of Arabization and even married into the local population, and their tribes were called “Arab al‐Turkeman” (http://www.alnssabon.com/t11489.html).

In family 2 the origin of the mother's variant could be either paternal or maternal (Figure 1A). Her paternal grandparents are related and belong to the Battat clan from the town of ad‐Dhahiriya in the southern Hebron hills. Some of its members settled from the 12th century in the city of Hebron as well. The Battat families see themselves as originating from the tribe of Quraysh and the house of Prophet Muhamad. These families have branches in both Egypt and Iraq (http://alsadaalalawenthiqar.rigala.net). The mother's maternal grandparents belong to the Rabba and Hawarin clans that maintain long lasting coalition with the Battat families and frequently intermarry with them.

In family 3, which is one of numerous Kabha families living in the region, the family tree (Figure 1A) suggests that the variant comes from the maternal side belonging to the Milhim clan. Because the Kabha and Milhim families are historically interrelated by multiple family ties we choose to investigate both of them. The Kabha families' origins are from the city of Ta'if located in the province of Hejaz, presently Saudi Arabia. It is recognized as originally affiliated with the Thaqif coalition comprised of the northern tribes in the Arabian Peninsula, also known as the “Qays” tribes. They arrived to Palestine in the seventh century as part of the Muslim conquests and settled in the Hebron hills, in the villages Yatta, Dura, ad‐Dhahiriya, and Bayt Jibrin. In the 16th century, once Ottoman rule of the land had been initiated, some of its members moved to the northern slopes of the Nablus hills and resided in the town of Ya'abad, from where they embarked westwards and founded seven small villages that in time became full villages. These are Tura, Bart'ah, Um al‐Qataf, Baydus, Wad 'Ara, 'Ayn al‐Sahala, and Khur Saqer,^15^ interview with 'Abad al‐Rahaman Dawoud Kabha, May 8, 2009). The Kabha families have marriage ties with all the other families in the region. Among others with the Turkmen tribes who settled in the area of Karkur, the Milhim families and with members of the Kabha families in the eastern suburbs of 'Ar'ara. The Milhim family that lived in the neighborhood of Wadi al‐Qasab is one of the founding families of 'Ar'ara in the 18th century. They came to 'Ar'ara from the town of Halhul in the Hebron hills, where one of the major families still carries the name Milhim. They are recognized as originally affiliated with the coalition of Yemenite (Southern) tribes which since the third century migrated and settled in the area of Wadi Sarhan and associated with the tribe of Sirhan (Sarahin) in the northern Arabian Peninsula.^16^

## 4 DISCUSSION

In this report we demonstrate that the c.2164A>T (Lys722Ter) variant of the *XDH* gene identified in a Turkmen family from Turkey^8^ and two Israeli Arab families is a common ancestral mutation. As far as we are aware this is the first description of an ancestral mutation causing classical xanhinuria. Among 21 distinct variants causing hereditary xanthinuria reported so far (Table 1) several were identified in more than one family (highlighted in Table 1). Yet all, except the c.2567delC and c.2164A>T variants in the *XDH* gene, occurred at mutational hot spots, for example, C<T transitions at CpG sites and deletions/insertions in mononucleotide tracks. Thus, these are most probably recurrent variants as was demonstrated by analysis of nearby polymorphisms in two cases^2^, ^7^ while in another case genetic tests failed to prove relatedness of the affected families.^23^ In our study of two Arab and a Turkmen family the c.2164A>T *XDH* gene variant was linked to an extended haplotype of polymorphic markers that was absent in 25 control chromosomes, demonstrating its common origin (Figure 2). We found that the MRCA of the three families is 179 (95% credible limit 70) generations old. Interestingly, the Turkmen family 1 and the Arab family 2 are more closely related than the Arab families 1 and 3 (Figure 2). The genealogical history of these families revealed their lengthy settlement in the Hebron hills and an extended geographical network of their family branches. Taken together with the general history of the region and the present day dispersion of the Turkmens and Arabs (see details in Supporting Information) these findings strongly suggest a wide dispersion of the *XDH* gene c.2164A>T variant in a region prone to urolithiasis, the Afro‐Asian stone‐forming belt.^5^ Our finding contributes to the view that hereditary xanthinuria might be not such a rare disease.^3^, ^32^, ^33^

**Table 1:** Variants reported in patients with classical xanthinuria

	Variant	Patients' origin	Reference
*XDH* gene			
1	**c.682C>T** a	Japan	1
	**p.Arg228Ter**	Japan	
2	**c.2567delC**	Japan	1
	**p.(Thr856fs)**	Japan	17
		Japan	18
3	c.1658insC p.(Ala556fs)	Israel (Iranian‐Jewish)	6
4	c.445C>T	Japan	19
	p.Arg149Cys		
5	**c.2164 A>T**	Turkey (Turkmen)	8
	**p.Lys722Ter**	Israel (Arab)	Peretz et al. (this report)
6	**c.2729C>T**	Armenia	20
	**p.Thr910Met**		
		Poland	21
		Afghanistan	22
7	**c.641delC**	Poland	21
	**p.(Pro214fs)**	Czech Republic	23
		Czech Republic	
		Germany	24
8	**c.141insG**	Afghanistan	22
	**p.(Cys48fs)**		
		UK‐Indian Subcontinent	25
9	c.2473C>T	Czech Republic	23
	p.Arg825Ter		
10	c.2641C>T	Czech Republic	23
	Arg 881Ter		
11	exon2‐4 del (~11 kbp)	Germany	26
12	c.3536T>C	France	27
	p.Ile1179Thr		
13	c.651+1G>T	France	27
14	c.3129‐3132del TCAT	France	27
	p.(His1044fs)		
15	c.3847C>T	Japan	28
	p.Arg1283Ter		
16	c.305A<G	Japan	18
	p.Gln102Arg		
*MOCOS* gene			
1	**c.1255C>T**	Japan	2
	**p.Arg419Ter**	Japan	
		China	29
2	c.169G>C	Japan	30
	p.Ala57Pro		
3	c.881C>T	Germany	31
	p.Thr294Ile		
4	**c.2326C<T**	Israel (Bedouin Arab)	7
	**p.Arg776Cys**	Israel (Italian Jewish)	
5	c.1034insA	Israel (Italian Jewish)	7
	p.(Ser344fs)		

a Same variants identified in apparently unrelated patients are highlighted by bold letters.

The biochemical hallmark of classical xanthinuria is very low to undetectable levels of UA and elevated levels of Xanthine and Hypoxanthine in blood and urine. A review of more than 150 cases of classical xanthinuria reported from 22 countries^3^, ^33^ shows that the disorder is partially penetrant regarding its clinical symptoms. Around 40% of the patients presented with symptoms that could be attributed to the defect: irritability, hematuria, urinary tract infection, renal colic, crystallurias, urolihiasis and acute renal failure. The rest were totally asymptomatic sibs (20%) or detected during population studies or during routine laboratory tests for presumably unrelated disorders. Symptoms may appear as early as the neonatal period^34^ or not appear at all until advanced age.^6^, ^7^ The clinical features of the six patients, bearing the c.2164 A>T *XDH* variant described in this investigation are consistent with this statistics. Three affected children in family 2 were asymptomatic and came to the attention of the treating physicians during the follow up of the index case for a neurogenic bladder. In family 1 both affected siblings were symptomatic. One presented at the age of 8 months with failure to thrive, vomiting, and discomfort during urination, gross hematuria and passage of stones. The older brother had bladder and kidney stones that were removed surgically at the age of 5 years.^8^ A very similar case of early onset (9 months) of severe symptoms of the disorder was reported from Turkey quite recently.^35^ The most severe presentation was in family 3 were the disorder progressed to a non‐functioning left kidney necessitating nephrectomy at the age of 11 years. An even more rapid progression of the disorder leading to nephrectomy in a 3 years old boy was recently reported from Tunisia.^36^ Another important health aspect of classical xanthinuria, irrespective of its clinical manifestation is the risk of drug toxicity/inefficiency due to the fact that both XDH and AO are drug‐metabolizing enzymes.^4^ Two cases of severe thiopurine‐induced toxicity in xanthinuria patients were recently reported.^37^, ^38^

We believe that our work will increase the awareness to classical xanthinuria among the Arab and Turkmen populations, will lead to identification of more cases allowing for further investigation of its dispersion and prevention of its potential severe, in some instances life threatening clinical and toxicological complications.

## CONFLICT OF INTEREST

H.P., M.K., D.M.S., M.K., S.U., I.K., H.S., D.La., and D.Le. declare that they have no conflict of interest.

## AUTHOR CONTRIBUTIONS

H.P. conceived, initiated, designed the experiments, analyzed the data, and wrote the initial version of the manuscript. M.K. and D.M.S. designed, performed, and wrote the text for estimation of MRCAs. M.K. researched and wrote the historical genealogy of the studied families. S.U. planned and performed the laboratory experiments. I.K., H.S., D.La, and D.Le. provided and wrote the text for demographic, family tree, and clinical data.

All coauthors read and approved the final version of the article.

## COMPLIANCE WITH ETHICAL STANDARDS

All procedures followed were in accordance with the ethical standards of the Helsinki Declaration of 1975, as revised in 2000. Informed consent was obtained from all patients for being included in the study and the study was approved by the Institutional Helsinki Committee of the Tel Aviv Sourasky Medical Center, approval number 0367‐08‐TLV.

## Supporting information


**Table S1.** Published physical and recombination map positions of polymorphic markers on chromosome 2p22‐23 and primers' sequences used for the PCRs
**Table S2.** Range and frequency of polymorphic alleles identified on control chromosomes
**Table S3.** Published and calculated genetic map positions and calculated distances to the site of the mutation of the polymorphic markers used in computation of the MRCAs
**Figure S1.** Genotyping of microsatellite markers (A) and segregation analysis of alleles (B) in family 2

## References

[1] Ichida K , Amaya Y , Kamatani N , Nishino T , Hosoya T , Sakai O . Identification of two mutations in the human xanthine dehydrogenase gene responsible for classical type I xanthinuria. J Clin Invest. 1997;99:2391‐2397. 9153281 10.1172/JCI119421 PMC508078

[2] Ichida K , Matsumura T , Sakuma R , Hosoya T , Nishino T . Mutation of human molybdenum cofactor sulfurase gene is responsible for classical xanthinuria type II. Biochem Biophys Res Commun. 2001;282:1194‐1200. 11302742 10.1006/bbrc.2001.4719

[3] Simmonds HA , Reiter S , Nishino T . Hereditary xanthinuria In: Scriver CR, Beaudet AL, Sly WS, et al., eds. The Metabolic and Molecular Bases of Inherited Diseases. New York: McGraw‐Hill; 1995:1781‐1797.

[4] Kitamura S , Sugihara K , Ohta S . Drug‐metabolizing ability of molybdenum hydroxylases. Drug Metab Pharmacokinet. 2006;21:83‐98. 16702728 10.2133/dmpk.21.83

[5] Lopez M , Hoppe B . History, epidemiology and regional diversities of urolithiasis. Pediatr Nephrol. 2010;25:49‐59. 21476230 10.1007/s00467-008-0960-5 PMC2778769

[6] Levartovsky D , Lagziel A , Sperling O , et al. XDH gene is the underlying cause of classical xanthinuria: a second report. Kidney Int. 2000;57:2215‐2220. 10844591 10.1046/j.1523-1755.2000.00082.x

[7] Peretz H , Shtauber‐Naamati M , Levartovsky D , et al. Identification and characterization of the first mutation (Arg776Cys) in the C‐terminal domain of the human molybdenum cofactor sulfurase (HMCS) associated with type II classical xanthinuria. Mol Genet Metab. 2007;91:23‐29. 17368066 10.1016/j.ymgme.2007.02.005

[8] Gok F , Ichida K , Topaloglu R . Mutational analysis of the xanthine dehydrogenase gene in a Turkish family with autosomal recessive classical xanthinuria. Nephrol Dial Transplant. 2003;18:2278‐2283. 14551354 10.1093/ndt/gfg385

[9] Litwin H . Methodological issues in measuring social networks (Hebrew). Gerontology. 2001;3/4(special issue):155‐168.

[10] Ihsan A‐N. The Book of Arab Genealogy, Damascus: Dar al‐Furqan; 2001.

[11] Agmon I . Family and Court: Legal Culture and Modernity in Late Ottoman Palestine. Syracuse: Syracuse University Press; 2005.

[12] Doumani B . Rediscovering Palestine: Merchants and Peasants in Jabal Nablus, 1700–1900. Berkeley and Los Angeles: University of California Press; 1995.

[13] Owen R . Studies in the Social and Economic History of Palestine in the Nineteenth and Twentieth Centuries. London: Macmillan; 1982.

[14] UNESCO Document 159759 ; June 2017 https://whc.unesco.org/document/159759. Accessed June, 2019.

[15] Kabha W . 'Asherat al‐Kabha Madin, Wa Hadir (the Kabha Family: Past and Present). Um al‐Fahem: Dar al‐Ma'rifah; 1996:19‐31.

[16] Aql M . Al‐Mufassal fi Tarikh Wadi 'ara (The wide history of Wadi 'ara) 'Ar'ara: Matba'at al‐Amal; 2004:161‐170.

[17] Fujiwara Y , Kawakami Y , Shinohara Y , Ichida K . A case of hereditary xanthinuria type I accompanied by bilateral renal calculi. Intern Med. 2012;51:1879‐1884. 22821105 10.2169/internalmedicine.51.6891

[18] Iguchi A , Sato T , Yamazaki M , et al. A case of xanthinuria type I with a novel mutation in xanthine dehydrogenase. CEN Case Rep. 2016;5:158‐116. 28508967 10.1007/s13730-016-0216-3 PMC5413754

[19] Sakamoto N , Yamamoto T , Moriwaki Y , et al. Identification of a new point mutation in the human xanthine dehydrogenase gene responsible for a case of classical type I xanthinuria. Hum Genet. 2001;108:279‐283. 11379872 10.1007/s004390100477

[20] Arikyants N , Sarkissian A , Hesse A , Eggermann T , Leumann E , Steinmann B . Xanthinuria type I: a rare cause of urolithiasis. Pediatr Nephrol. 2007;22:310‐314. 17115198 10.1007/s00467-006-0267-3

[21] Jurecka A , Stiburkova B , Krijt J , Gradowska W , Tylki‐Szymanska A . Xanthine dehydrogenase deficiency with novel sequence variations presenting as rheumatoid arthritis in a 78‐year‐old patient. J Inherit Metab Dis Suppl. 2010;3:S21‐S24. 10.1007/s10545-009-9011-z 20077140

[22] Nakamura M , Yuichiro Y , Oliver SJ , et al. Identification of a xanthinuria type I case with mutations of xanthine dehydrogenase in an Afghan child. Clin Chim Acta. 2012;414:158‐160.22981351

[23] Stiburkova B , Krijt J , Vyletal P , et al. Novel mutations in xanthine dehydrogenase/oxidase cause severe hypouricemia: biochemical and molecular genetic analysis in two Czech families with xanthinuria type I. Clin Chim Acta. 2012;413:93‐99. 21963464 10.1016/j.cca.2011.08.038

[24] Darr RW , Lenzner S , Eggermann T , Darr WH . Xanthinuria type 1 in a woman with arthralgias: a combined clinical and molecular genetic investigation. Dtsch Med Wochenschr. 2016;141:571‐574. 27078247 10.1055/s-0041-106053

[25] Stockdale C . A Rare Cause of Renal Stone Formation in Two Siblings; 2013 http://www.acb.org.uk/docs/default/source/committees/regions/sww/trainingday180714-renal-stone-case-cs907C02B2FFB5.pdf?sfvrsn=4. Accessed June, 2019.

[26] Eggermann T , Spengler S , Denecke B , Zerres K , Mache CJ . Multi‐exon deletion in the *XDH* gene as a cause of classical xanthinuria. Clin Nephrol. 2013;79:78‐80. 23249873 10.5414/cn106994

[27] Diss M , Ranchin B , Broly F , Pottier N , Cochat P . Type I xanthinuria: report on three cases. Arch Pediatr. 2015;22:1288‐1291. 26521682 10.1016/j.arcped.2015.09.003

[28] Tanaka KI , Kanazawa I , Yamasaki H , Hasegawa H , Ichida K , Sugimoto T . Xanthinuria type I with a novel mutation of xanthine dehydrogenase. Am J Med Sci. 2015;350:155‐156. 26110747 10.1097/MAJ.0000000000000498

[29] Zhou Y , Zhang X , Ding R , et al. Using next‐generation sequencing to identify a mutation in human MCSU that is responsible for type II xanthinuria. Cell Physiol Biochem. 2015;35:2412‐2421. 25967871 10.1159/000374042

[30] Yamamoto T , Moriwaki Y , Takahashi S , et al. Identification of a new point mutation in the human molybdenum cofactor sulfurase gene that is responsible for xanthinuria type II. Metabolism. 2003;52:1501‐1504. 14624414 10.1016/s0026-0495(03)00272-5

[31] Finckh U , Haddad M , Lukacs Z , Wagener C , Gal A . Clasical Xanthinuria Type 2 Associated with a Missense Mutation in HMCS, the Gene Encoding Molybdenum Cofactor Sulfurase. Clin Chem Lab Med. 2003; 41:A61‐A‐129.

[32] Sebesta I , Stiburkova B , Krijt J . Hereditary xanthinuria is not so rare disorder of purine metabolism. Nucleosides Nucleotides Nucleic Acids. 2018;37:324‐328. 29723117 10.1080/15257770.2018.1460478

[33] Simmonds HA . Hereditary Xanthinuria. Orphanet Encyclopedia; July 2003 http://www.orpha.net/data/patho/GB/uk-XDH.pdf. Accessed June, 2019.

[34] Badertscher E , Robson WLM , Leung AKC , Trvenen CL . Xanthine calculi presenting at 1 month of age. Eur J Pediatr. 1993;152:252‐254. 8444255 10.1007/BF01956156

[35] Akinci N , Cakil A , Oner A . Classical xanthinuria: a rare cause of pediatric urolithiasis. Turk J Urol. 2013;39:274‐276. 26328123 10.5152/tud.2013.066 PMC4548614

[36] Bahlous A , M'rad M , Kalaie E , et al. Hereditary xanthinuria with recurrent urolithiasis occurring in infancy. Gene Technol. 2016;5:139 10.4172/2329-6682.1000139

[37] Stiburkova B , Pavelcova K , Petru L , Krijt J . Thiopurine‐induced toxicity is associated with dysfunction variant of the human molybdenum cofactor sulfurase gene (xanthinuria type II). Toxicol Appl Pharmacol. 2018;353:102‐108. 29935280 10.1016/j.taap.2018.06.015

[38] Tanev D , Peteva P , Fairbanks L , et al. Beware of the uric acid: severe azathioprine myelosuppression in a patient with juvenile idiopathic arthritis and hereditary xanthinuria. J Clin Rheumatol. 2018 [Ahead of Print]. 10.1097/RHU.0000000000000838. Accessed June, 2019. 32073534

