# Supplementary material for: An ancestral variant causing type I xanthinuria in Turkmen and Arab families is predicted to prevail in the Afro‐Asian stone‐forming belt
Source: JIMD Rep. 2019 Dec 5;51(1):45–52. doi: 10.1002/jmd2.12077 (PMC7012738; doi:10.1002/jmd2.12077)
Supplement: Supplementary file 1 — Table S1. Published physical and recombination map positions of polymorphic markers on chromosome 2p22‐23 and primers' sequences used for the PCRs Table S2. Range and frequency of polymorphic alleles identified on control chromosomes Table S3. Published and calculated genetic map positions and calculated distances to the site of the mutation of the polymorphic markers used in computation of the MRCAs Figure S1. Genotyping of microsatellite markers (A) and segregation analysis of alleles (B) in family 2 [file JMD2-51-45-s001.docx]

**Supplementary Material**

**Methods**

*Model for estimation of the time to the Most Recent Common Ancestor (MRCA)*

Our estimation of the time to the MRCA applies Bayesian methods to a simple model of crossover events through generations in the chromosomes carrying the inherited mutation. The model relates the length *U* (in Morgans) of the intact interval (on one side of the mutation site) to the number *T* of generations between the common ancestor and the recently selected carriers. Observations are limited to a small set of genomic markers (Table S1), so that we actually observe *u_1_*, the longest distance with no recombinant events, and *u_2_*, the distance to the marker that exhibits the first recombination event (Figure 2). Thus *u_1_* < *U* < *u_2_*. The probability that, in *T* generations, there are no recombination events in the interval to u_1_ and at least one such event in the interval to u_2_ is given by

$$P\left( u_{1}<U<u_{2} \right)={(1-p)}^{2T}[1-{(1-q)}^{2T}]$$

Where $p=1-exp(-u_{1})$ and $q=1-exp(-\left( u_{2}-u_{1} \right))$ are the per generation probabilities of at least one recombination event in the intervals (*0,u_1_*) and (*u_1_,u_2_*), respectively. The above term is the likelihood contribution (as a function of *T*), from the observed interval. If data are available for both telomeric and centromeric sides from the mutation, each of them contribute an interval and the complete likelihood for *T* is the product of the two individual likelihood contributions.

We adopt a uniform prior distribution for *T* up to a maximum of 500 generations (i.e., about 10,000 years). We use the mode of the resulting posterior distribution as a point estimate of *T*. For our prior, this coincides with the maximum likelihood estimator. In assessing the uncertainty of our estimate, we are most interested in establishing a minimal number of generations to the MRCA. Thus we compute a one-sided 95% credible interval (the Bayesian equivalent of a confidence interval) with a lower limit only, which is given by the 0.05 quantile of the posterior distribution.

The model application assumes that the ancestral allelic sequence is rare among non-carrier haplotypes. Possible recombination between carrier chromosomes and a possible mutation in the marker alleles was ignored.

**Discussion**

*Summary of the history and present day dispersion of the Turkmen and Arab peoples*

**The Turkmen** speak an Oghuz language originating from a long lasting Turkic-Mongolian linguistic unity present around 4500–4000 BC in Southern Siberia and Mongolia. Recent genetic studies confirm their Asian ancestry in Southern Siberia and Mongolia [Yunusbayev et al 2015, Petit et al 2019]. This region belongs to the Euro-Asian Steppe belt that since the Paleolithic Age has connected Eastern Europe, Central Asia, China, South Asia and the Middle East economically, politically, and culturally through overland trade routes that gradually developed to become part of the famous "Silk Roads" [<https://en.wikipedia.org/wiki/Eurasian_Steppe>]. Numerous migrations and invasions of Turko-Mongol among other nomadic tribes gravitated toward the ancient settled regions of the South including Mesopotamia, Elam and the Indus Valley civilization [<https://en.wikipedia.org/wiki/History_of_Turkmenistan>]. In the Middle Ages many of the Turkish nomads were mercenaries in the employ of local Arab and Persian rulers and some of them assimilated into the local populations [<https://en.wikipedia.org/wiki/Iraqi_Turkmen>, Pillalamarri 2016]. In the 9^th^ and 10^th^ centuries Oghuz military expansions in the area between the Caspian and Aral Seas reached the periphery of the Muslim world. At the beginning of the 11^th^ century an Oghuz clan leader named Seljuk founded a dynasty and an empire on the basis of those Oghuz elements that had migrated southward into present day Turkmenistan and Iran [<https://www.newworldencyclopedia.org/entry/Seljuk_Turks>]. The name "Turkmen" was coined in the 10^th^ century to distinguish those Oghuz groups who migrated south into the Seljuk domains and accepted Islam from those that had remained in the steppe [Curtis 1996]. The Seljuk Empire declined at the end of the 12^th^ century, the Seljuk clans established small principalities throughout Anatolia that, for a short time, became vassals of the Mongol conquerors and became again independent after the Mongol withdrawal. Legend says that the *bey* (chief) of a small tribe that extended from the **Kayi** branch of the Seljuk Turks (the origin of **Family 1**) in western Anatolia helped Osman to found the Ottoman Empire (1299-1922) [<https://en.wikipedia.org/wiki/Sogut>]. At its height the Ottoman Empire encompassed most of southeastern Europe and parts of Ukraine, portions of the Middle East (Iraq, Syria, Palestine and Egypt), North Africa and large parts of the Arabian Peninsula [Yapp and Shaw 2018]. The present day homeland of the Turkmen is Turkmenistan located on the eastern side of the Caspian Sea, nevertheless clusters of Turkmen populations are scattered in neighboring parts of Central Asia (North of Iran and Afghanistan). Further pockets of Turkmen are found in the Middle East (Iraq, Syria, Israel, Palestine and Lebanon) and smaller groups live in central Turkey [Curtis 1996, Hurmuzlu 2015].

**The Arabs** are a people of various ancestral origins, religious backgrounds and history who became to share common cultural traits including the Arabic language [Nebel et al 2002, Tadmouri et al 2014]. Arabic is a Semitic language belonging to the Afro-Asian language family. One study suggests that all known Semitic languages can be traced back to a Proto-Semitic language in the Levant to around 3750 BC, that around 800 BC was introduced from South Arabia into the Horn of Africa [<https://en.wikipedia.org/wiki/Proto-Semitic_language>]. Genetic studies as well as archeologic findings support the important role of the Levant in shaping the Neolithic dispersal of human settlements in the Arabian Peninsula. It was found that approximately 62–69% of today’s males in Saudi Arabia share common genetic structures with those in the Near East [Tadmouri et al 2014]. According to archeological findings [Metropolitan Museum of Art, 2000] the earliest people entered the western Arabian Peninsula from the Levant by the 8^th^ millennium BC and between 6000–2000 BC Stone Age and Bronze Age inhabitants setup intricate trade routes between Arabia, Mesopotamia and the Indus Valley. By 5000 BC settlements appear in the east coast of the Peninsula, as south as Oman, with features similar to those of the Levant and southern Mesopotamia. Starting from the 4^th^ millennium BC onward the people living in the Peninsula developed a variety of distinct societies: nomadic tribes, sedentary farmers in large oasis, self-governing cities, merchant colonies within cities and kingdoms of various sizes [Hoyland 2002, McDonald et al 2013]. They maintained extensive terrestrial and maritime trading within the Peninsula and outside of it from Egypt, through the Levant to Mesopotamia, Iran and as far as India and Africa. Some of them were also renowned fighters providing military manpower experienced in travel in the desert for the rivaling powers in their vicinity. The "Arabs" are first mentioned in Biblical and Assyrian texts of the 9^th^ to 5^th^ centuries BC and ancient sources including those of the Assyrians and Babylonians and later the Persians, Greeks and Romans defined "Arabia" as anywhere inhabited by peoples called Arabs: the Nile delta and Eastern Egypt, the Sinai Peninsula, Gaza, south of Palestine-the Negev, todays Jordan, Syria, Lebanon, south Mesopotamia, parts of the Arabian Peninsula, the head of the Persian Gulf and even Central Iran. Persian explorers of the 3^rd^ century BC were the first to define the Peninsula as "Arabian". Yet, only around the first century BC "Arabization" of the whole Peninsula, including a process of absorption of the cultural traditions of the South and East Arabia led to the gradual transformation of the originally diverse peoples of Arabia into a single ethnic group [Hoyland, 2002]. In the 7^th^ to 9^th^ century the Islamic religion spread across the Peninsula and an Islamic Empire that extends from Spain to India was established. Between 850–1300 the Empire declined, Arabia's old trade routes collapsed and small sheikhdoms came under the control of Tartar moguls, Persians and Ottoman Turks. From 1517 to 1918 much of the Arab world was under the suzerainty of the Ottoman Empire. Today, Arabic populations inhabit 23 formal Arab States located in North and North-East of Africa, the Middle East and the Arabian Peninsula and some other places like Southern Iran, Turkey and Israel [Tadmouri et al 2014].

**Table S1.** Published physical and recombination map positions of polymorphic markers on chromosome 2p22-23 and primers' sequences used for the PCRs

| Marker | Locus/gene | Map position^1^ | | Primers' sequence |
| --- | --- | --- | --- | --- |
|  |  | bp | cM  (deCode) |  |
| M1 | D2S390 | 29,788,997 | 52.47 | F:5'-CCAGTTCTCTAACCAATGTC  R:5'-GAGATTCTCAGCATTTGCTC |
| M2 | D2S400 | 30,920,525 | 54.55 | F:5'-GACTGAACAAACCATGTTTAGG  R:5'-GCTAAGATAATCTCCCTGAG |
| M3 | D2S2255 | 31,000,369 | - | F:5'-GTCTCCAGGCTACTTTGAGG  R:5'-CTTGCTTGTCTCAGGTCATG |
| M4 | D2S2283 | 31,211,646 | - | F:5'-ATAGCGACTCAGGTTACTAG  R:5'-CCAGGTAACCACTGACTTGC |
| M5 | D2S352 | 31,278,292 | 55.21 | F:5'-GCAGAGGCACTTTTCAATGTC  R:5'-TGGCCAAAGTATTGGGAACAC |
| m1 | XDH c.2197+68G/A | 31,367,821 | - | F:5'-GAGTCAACAATTCAGAGAGC  R:5'-ACTTCCCTGCTTCAGGGTTC |
| m2 | XDH c.2197+42G/C | 31,367,922 | - | Idem |
| **m** | XDH **c.2164A>T*** | 31,367,994 | - | Idem |
| m3 | XDH c.2107A/G | 31,368,051 | - | Idem |
| M6 | D2S2203 XDH IVS9 | 31,383,961 | 55.37 | F:5'-TAGTACCTCATAGGGTTGTTG  R:5'-GTCTGGGAATAGGAAACTCAG |
| M7 | D2S2351 | 31,861,746 | - | F:5'-CGGTGTTTAGATTCTCTTTGG  R:5'-TTGCCTATCCTGTCTTCAAG |
| M8 | D2S2325 | 32,930,388 | - | F:5'-TAATTAGATTCAGCCTCCTTC  R:5'-GATAACCACGTGATCAAAGAC |
| M9 | D2S2347 | 33,151,729 | 56.05 | F:5'-GTCACAACTGAAAATTGTGG  R:5'-TCTCCAGTCTCATCTATGTG |
| M10 | D2S367 | 34,216,081 | 57.89 | F:5'-ATTATGGAGTTGGCCACTGC  R:5'-GCTTCTTGTTCACAGGTGTG |

^1^ <https://www.ncbi.nlm.nih.gov/> assembly GRCh38.p7, annotation release 108, *rs72549367

PCRs were performed on a PTC100 Programmable Thermal Controller (MJ Research Inc.) with the following program: 95^0^C 5', [95^0^C 1' 60^0^C 1' 72^0^C 1']X30, 72^0^C 5', 15^0^C Hold.

**Table S2.** Range and frequency of polymorphic alleles identified on control chromosomes

| Marker | Apparent allele size (bp) | | |
| --- | --- | --- | --- |
|  | Range | Most frequent (frequency, n) | Conserved on mutated chromosomes (frequency, n) |
| M1 | 381-411 | 407 (0.33, n=21) | - |
| M2 | 307-321 | 321 (0.48, n=23) | - |
| M3 | 253-269 | 253 (0.33, n=6) | - |
| M4 | 394-405 | 403 (0.23, n=26) | 401 (0.19, n=26) |
| M5 | 294-312 | 308 (0.28, n=25) | 298 (0.12, n=25) |
| m1 | G/A | G (0.71, n=28) | A (0.29, n=28) |
| m2 | G/C | G (0.79, n=28) | G (0.79, n=28) |
| m3 | A/G | A (0.86, n=28) | A (0.86, n=28) |
| M6 | 272-286 | 282 (0.38, n=24) | 282 (0.38, n=24) |
| M7 | 427-435 | 431 (0.30, n=23) | 432 (0.13, n=23) |
| M8 | 354-366 | 356 (0.33, n=26) | 362 (0.08, n=26) |
| M9 | 166-186 | 178 (0.15, n=26)  182 (0.15, n=26) | - |
| M10 | 270-295 | 293 (0.40, n=25) | - |

**Table S3.** Published and calculated* genetic map positions and calculated distances to the site of the mutation of the polymorphic markers used in computation of the MRCAs

| Marker | Position  ( cM) | Distance from the mutation (M) |
| --- | --- | --- |
| M3 | 54.70* | 0.006485291 |
| M4 | 55.09* | 0.002587703 |
| M5 | 55.21 | 0.001358234 |
| **m** | 55.35* | 0 |
| M7 | 55.55* | 0.002079642 |
| M8 | 55.96* | 0.006190343 |
| M9 | 56.05 | 0.007041723 |

*****Estimated based on published physical and recombination map positions of flanking markers (Table S1) under the assumption of linear proportionality.

**Figure S1**. Genotyping of microsatellite markers (**A**) and segregation analysis of alleles (**B**) in Family 2

1. Chromatograms showing fragment sizes of 10 microsatellite markers (M1-M10) obtained in the DNA sample of the carrier mother of Family 2. The LIZ600 molecular weight marker (Applied Biosystems, USA) was used to calibrate the system and the GeneMapper 4.0 software for fragments' size analysis
2. SNP alleles are designated by the observed nucleotides' symbols while alleles of the microsatellite markers are represented by the observed sizes (bp) of the amplified fragments. The haplotype associated with the maternal mutated chromosome is framed.

**References**

Curtis GE 1996 (ed) Turkmenistan: A Country Study (chapters: "Oghuz and the Turkmen", "Seljuk Period", "Formation of the Turkmen Nation"). Washington: GPO for the Library of Congress <http://countrystudies.us/turkmenistan/>

Hoyland RB (2002) Arabia and the Arabs: From the Bronze Age to the coming of Islam, Taylor & Francis e-Library <http://www.almuslih.org/Library/Hoyland,%20R%20-%20Arabia%20and%20the%20Arabs.pdf>

Hurmuzlu E (2015) The Turkmens of the Middle East. Turkish Policy Quarterly 14(1) 1-9 <http://turkishpolicy.com/Files/ArticlePDF/the-turkmens-of-the-middle-east-spring-2015-en.pdf>

McDonald M, Nebes N, Fisher G, translations by Klein K (2013) Ancient Arabia: A brief history and timeline.

<http://krc.orient.ox.ac.uk/resources/publications/macdonald/Ancient%20Arabia%20a%20Brief%20History%20and%20Time-Line.pdf>

Metropolitan Museum of Art (New York) 2000, Heilbrunn Timeline of Art History: Arabian Peninsula, 8000-2000 BC <https://www.metmuseum.org/toah/ht/02/wap.html>

Nebel A, Landau-Tasseron E, Filon D, Oppenheim A, Faerman M (2002) Genetic Evidence for the Expansion of Arabian Tribes into the Southern Levant and North Africa. Am J Hum Genet 70: 1594–1596

Petit F, Minnai F, Chiaroni J et al (2019) The radial expansion of the Diego blood group system polymorphisms in Asia: mark of co-migration with the Mongol conquests. Eur J Hum Genet 27: 125–132

Pillalamarri A (2016) The epic story of how the Turks migrated from Central Asia to Turkey. <https://thediplomat.com/2016/06/the-epic-story-of-how-the-turks-migrated-from-central-asia-to-turkey/>

Tadmouri GO, Sastry KS, Chouchane L (2014) Arab gene geography: from population diversities to personalized medical genomics. Global Cardiol Sci Pract 54: 393-408 <http://dx.doi.org/10.5339/gcsp.2014.54>

Yapp ME and Shaw SJ (2018) Ottoman Empire, Historical Empire, Eurasia and Africa. <https://www.britannica.com/place/Ottoman-Empire>, last updated Aug 17, 2018

Yunusbayev B, Metspalu M, Metspalu E et al (2015) The genetic legacy of the expansion of Turkic-speaking nomads across Eurasia. PLoS Genet 11: e1005068. doi:10.1371/journal.pgen.1005068
